# Description of clinical cases and available diagnostic tools of oropharyngeal syphilis: a systematic review of the literature

**DOI:** 10.1186/s12879-024-10129-1

**Published:** 2024-11-06

**Authors:** Pierre Guarino, Francesco Chiari, Carlo Carosi, Giustino Parruti, Claudio Donadio Caporale, Livio Presutti, Gabriele Molteni

**Affiliations:** 1 Otolaryngology Head and Neck Unit - “Santo Spirito” Hospital, Pescara, Italy; 2 Otolaryngology and Audiology, IRCCS Azienda Ospedaliero-Universitaria di Bologna, Bologna, Italy; 3 Infectious Diseases Unit - “Santo Spirito” Hospital, Pescara, Italy; 4 Department of Medical and Surgical Sciences, Alma Mater Studiorum, University of Bologna, Bologna, Italy

**Keywords:** Syphilis, Oropharynx, Tonsil, Base tongue

## Abstract

**Introduction:**

Syphilis is a systemic bacterial infection caused by the spirochete *Treponema pallidum*. Head and neck mucosal manifestations of syphilis can be observed in each and all of primary, secondary and tertiary syphilis, especially in the secondary one. Therefore, oropharynx is an unusual localization of syphilitic lesions, mainly represented by ulcerous lesions, tissue hypertrophy, mucosal patches and cancer-like lesions. Serology is routinely considered the gold standard for the screening and diagnosis of syphilis. However, direct detection is routinely used during polymerase chain reaction (PCR) of oropharyngeal tissue and suspicious cervical lymphadenopathies.

**Methods:**

PRISMA 2020 guidelines were applied to make a systematic literature review with the aim to make an overview of clinical manifestations and diagnostic tools of oropharyngeal syphilitic infection. A computerized MEDLINE search was performed using the PubMed, Web of Science and Cochrane databases.

**Results:**

The intended analysis was based on 38 papers, including a total of 55 cases. The main localization of oropharyngeal infection was the tonsil (71%), followed by lateral and posterior wall of oropharynx (16%). Ulcerous lesions were the most frequently encountered lesions in the primary syphilis (56%) and secondary syphilis (36%), whereas gumma’s lesions were encountered in the tertiary syphilis (57%). Diagnosis based on serological assays was used in combination with non-treponemal methods to determine disease activity (80% cases).

**Conclusions:**

Oropharyngeal syphilis has historically been referred to as the “great imitator” due to its highly variable manifestations, which can resemble malignancies. Physicians have to recognize oropharyngeal luetic features early, in order to set up an effective diagnostic and therapeutic work-up.

## Introduction

Syphilis is a systemic bacterial infection caused by the spirochete *Treponema pallidum*, which can be transmitted via direct close contact with eroded syphilitic lesions, via blood and diaplacental. However, sexually transmission is the most common route [1]. The incidence dramatically decreased following the successful introduction of penicillin treatment in the middle of the 20th century; syphilis, however, re-emerged since the 1990s, mostly in populations of men who have sex with men (MSM), especially those co-infected with human immunodeficiency virus (HIV) and genital herpes virus infection [2]. As the disease is highly contagious, factors influencing syphilis might be the primary factors (and risk factors) in developing the infectivity. It depends on several elements, first of all the local and systemic immunity of the host. Undomiciled housing status, history of HIV, history of tobacco use, and history of drug misuse were identified as common risk factors related to syphilis [3].

The course of syphilis is distinguished into primary, secondary and tertiary syphilis [4]. During the primary phase, one or more sores (chancres) form at the site of bacterial entrance within 3 weeks after exposure [4]. Systemic symptoms of malaise, weight loss and fever indicate the progression to the secondary syphilis, which it is characterized by a disseminated rash over trunk, palms of hands and soles of feet, appearing 2 to 12 weeks after the chancre and sometimes before its healing [4]. The secondary syphilis is usually followed by a latent stage, lasting sometimes over 2 years, whereby the disease does not clinically manifest. When it becomes again symptomatic, ushering the tertiary phase, it can involve the central nervous system (neurosyphilis), skin, bone and the cardiovascular system [5]. Head and neck mucosal manifestations of syphilis can be seen in all syphilitic diseases, and especially in the second one [6]. Oral cavity and oropharynx are the most commonly involved head and neck sites [6]. Therefore, oropharynx is an unusual localization of syphilitic lesions, such as ulcerous lesion, tissue hypertrophy, mucosal patches and cancer-like lesions, rather than oral cavity that is more frequently involved [7]. Therefore, oropharyngeal lesions were clinically similar to tumoral ones causing a high risk of a wrong diagnosis. For this reason, distinguishing syphilis from carcinoma is especially relevant today, given the rising rates of spirochete infection and the current epidemic of papillomavirus (HPV) related oropharyngeal squamous cell carcinoma (OPSCC). Historically, serology is routinely considered the main diagnostic method, but it continues to have open issues, such as the lack of specificity of non-treponemal tests and the lack of correlation of treponemal tests with disease activity. Nowadays, direct detection of Treponema pallidum continues to evolve from microscopic examination of material from lesions for visualization of Treponema pallidum to molecular detection of the organism, according to CDC 2024 recommendations [8].

The present systematic review has the aim to make an overview of clinical manifestations and diagnostic tools of oropharyngeal syphilitic infection.

## Materials and methods

### Search strategy and information sources

PRISMA 2020 guidelines were applied in this systematic literature review [9]. A computerized MEDLINE search was performed using the PubMed service of the U.S. National Library of Medicine (www.pubmed.org), Scopus database (www.scopus.com) and Cochrane Database (www.cochrane.com) for articles published from 1971 up to 2024, running the search string reported in Table 1.

**Table 1:** Search string for each database

DATABASE	SEARCH STRING	ARTICLES FOUND
EMBASE/PUBMED	(oropharyngeal OR tonsil OR base tongue OR pharyn*) AND (syphilis)	319
SCOPUS	(oropharyngeal OR tonsil OR base tongue OR pharyn*) AND (syphilis)	72
COCHRANE	(oropharyngeal OR tonsil OR base tongue OR pharyn*) AND (syphilis)	6

### Study selection and data extraction

After processing the above selected databases by running the associated search engine set with the respective search strings (Table 1) in July 2024, the output abstracts and titles were screened independently by two of the authors (P.G., and F.C.), who subsequently met and discussed disagreements on citation inclusion. Inclusion criteria for abstract selection were the use of the English language and reporting of patients with oropharyngeal infection. Authors excluded studies with unavailability of abstracts, languages other than English, not describing clinical and diagnostic data of patients affected by oropharyngeal syphilis. Full texts were identified by the same criteria. A further manual check of the references included in the articles was performed.

### Quality assessment

Two authors (P.G. and F.C.) independently assessed the quality of the included studies using an assessment tool for case series and case reports [10], which considers 4 domains (selection, ascertainment, causality, and reporting) and provides 8 questions to aid a quality score. Studies were rated as having a low, moderate, or high risk of bias, according to the description thereof (Table 2). The articles with a high risk of bias were excluded from the analysis.

**Table 2:** Tool for evaluating the methodological quality of case reports and case series. The eight questions included in the evaluation tool are categorized into the following four domains: selection, ascertainment, causality and reporting. Within each domain the authors evaluated wheter the question related conditions were satisfied (1) or not (0). Final results differentiated case reports or series into 3 categories of risk of bias as follows: low risk (1) if all questions of the four domains were matched; intermediate risk (2) if questions of three out of the four domains were matched; high risk (3) if questions of less than three out of the four domains were matched

Authors	Year	Selection	Ascertainment	Causality	Reporting	Risk of bias
**Vincenti**	1971	1	1	1	1	**1**
**Fiumara**	1974	1	1	1	1	**1**
**Cohen**	1976	1	1	1	1	**1**
**Viers**	1981	1	1	0	1	**2**
Fiumara *	1982	1	1	0	1	**2**
**Baarsma**	1985	1	1	1	1	**1**
**Shimizu**	1989	1	1	1	1	**1**
**Ishimaru**	1997	1	1	1	1	**1**
**Mannarà**	1999	1	1	1	0	**2**
**Ditzen**	2005	1	1	1	1	**1**
**Hamlyn**	2006	1	1	1	1	**1**
**Oddò**	2007	1	1	1	1	**1**
**Ikenberg**	2010	1	1	1	0	**2**
**Gedela**	2012	1	1	1	1	**1**
**Ablanedo**	2013	1	1	1	1	**1**
**Drago**	2014	1	1	0	1	**2**
**Barbee**	2015	1	1	1	1	**1**
**Berezo**	2015	1	1	1	1	**1**
**Kollipara**	2015	1	1	0	1	**2**
**Petrich**	2015	1	1	1	1	**1**
**Shinkuma**	2015	1	1	1	1	**1**
**Smith**	2015	1	1	1	1	**1**
**Zhanli Fu**	2015	1	1	1	0	**2**
**Plana Pla**	2016	1	1	0	1	**2**
**Ong**	2017	1	0	0	0	**3**
**Komeno**	2018	1	1	1	0	**2**
**Sakthivel**	2018	1	1	0	1	**2**
**Brendel**	2019	1	1	1	1	**1**
**Jategaonkar**	2019	1	1	0	1	**2**
**Maki**	2021	1	1	1	1	**1**
**Mishal**	2021	1	1	0	1	**2**
**Camps**	2021	1	1	1	1	**1**
Camps *	2021	1	1	1	1	**1**
**Christmann**	2022	1	1	1	1	**1**
**Han**	2022	1	1	1	1	**1**
**Wu**	2022	1	1	0	1	**1**
**Culbert**	2023	1	1	0	1	**2**
**Sun**	2024	1	1	1	1	**1**
**Taniguchi**	2024	1	1	1	1	**1**

* = second paper

### Data analysis

Patients’ level data were extracted and summarized. Categorical variables were presented as frequency and percentage. Continuous variables were presented as mean and range. The statistical analyses were carried out by means of STATA v.14 (StataCorp LLC, College Station, TX, USA).

### Aims of the systematic review

This systematic literature review aimed to better characterize clinical manifestation and diagnostic features of syphilitic oropharyngeal lesions. The first focus was the description of specific lesions and clinical manifestations in patients affected by oropharyngeal syphilis. The second goal was an overview on the role of laboratory tests, histological examination and radiological scans to screen and diagnose syphilitic lesions.

### Informed consent and human and/or animal experimentation guidelines

An informed consent and human and/or animal experimentation guidelines in not indicated.

## Results

Running the search engine by setting the strings reported in Table 1, a total of 319, 72 and 6 manuscripts were identified in Embase/PubMed, Scopus and Cochrane databases, respectively. After removal of duplicates, 370 papers were selected. After filtering upon the abstract criterion, 112 articles were selected for full text screening. The other 258 articles were excluded due to unavailability of abstracts (10), language other than English (18), or not describing not describing clinical and diagnostic data of patients affected by oropharyngeal syphilis (230). Seventy-three full texts were excluded due to unavailability (4), not describing cases of oropharyngeal syphilis (7), or lacking data on gender, sex, site of infection, type of syphilis, symptoms and signs and laboratory exams, histological features or radiological findings (62). Finally, full texts were processed for risk-of-bias evaluation: as a result, one additional article was excluded for high risk of bias (Table 2). Therefore, the final analysis was carried out on 38 papers (20 case reports and 18 case series), which are indeed included in this review, carrying a low risk of bias (25) or an intermediate risk of bias (13), as reported in Table 2. The selected papers were published between 1971 and 2024. The total amount of patients included was 55. The largest study population included consisted of 6 patients.

### Epidemiological data and clinical manifestation of oropharyngeal syphilis

The entire cohort of patients included 47 (85.5%) males and 8 (14.5%) females. Mean age was 38.4 years (range 17–67 years). Sites of oropharyngeal infection were represented by tonsil (71% monolateral, 29% bilateral), lateral and posterior wall of oropharynx and base of tongue in 39 (71%), 9 (16%) and 7 (13%) patients, respectively. Secondary syphilis is the most frequent stage of syphilis was the second in 26 (48%) patients, followed by first in 22 (40%) patients, whereas a tertiary syphilis was diagnosed in 7 (12%) patients. Risk factors associated to oropharyngeal syphilis were reported in 37 cases, such MSM, high risk sexual behavior, smoke, HIV co-infection and drug misuse in 18 (49%), 10 (27%), 9 (24%), 9 (24%) and 1 (3%) patient, respectively. Oropharyngeal infection was accompanied by symptoms and signs such as sore throat (34 cases), skin papular rash (16 cases), neck mass (14 cases), odynophagia (5 cases), fever (4 cases), weight loss (3 cases), dysphonia (3 cases), and fatigue (3 cases), as detailed in Table 3. Compared in Table 4 are the rates of pathognomonic lesions among primary, secondary and late-stage syphilis. Ulcerous lesion (45%) were the most common lesions in patients affected by primary, secondary and late-stage syphilis, in 56%, 36% and 43% of cases, respectively. Other reported oropharyngeal signs of primary syphilis were tissue hypertrophy (22%) and mucosal patches (13%). Instead, secondary oropharyngeal syphilis manifested through tissue hypertrophy (28%), mucosal patches (8%) and hyperemia (8%). Finally, patients affected by late stage syphilis exclusively presented two lesions: gumma’s lesions (57%) and ulcerous lesions (43%).

**Table 3:** Cohort’s features

Variable		Nr. cases (range/perceptual)
Patient features	Mean age: years (range)	38.4 (17–67)
	Males: nr pts (%)	47 (85.5%)
	Females: nr pts (%)	8 (14.5%)
Site of infection	Tonsil: nr (%)	39 (71%)
	- monolateral side	27 (71%)
	- bilateral side	8 (29%)
	Lateral and posterior wall of oropharynx: nr (%)	9 (16%)
	Base tongue: nr (%)	7 (13%)
Type of syphilis	Primary: nr (%)	22 (40%)
	Secondary: nr (%)	26 (48%)
	Third: nr (%)	7 (12%)
Risk factors	MSM: nr (%)	18 (49%)
	High risk sexual behaviour*: nr (%)	10 (27%)
	Smoker: nr (%)	9 (24%)
	HIV: nr (%)	9 (24%)
	Drug: nr (%)	1 (3%)
Symptoms and signs	Sore throat: nr (%)	34 (65%)
	Rash-papular eruption: nr (%)	16 (31%)
	Neck mass: nr (%)	14 (27%)
	Odynophagia: nr (%)	5 (10%)
	Fever: nr (%)	4 (8%)
	Weight loss: nr (%)	3 (6%)
	Dysphonia: nr (%)	3 (6%)
	Fatigue: nr (%)	3 (6%)
Laboratorial tests and histological examinations	Surgical biopsy of oropharyngeal tissue: nr (%)	21 (38%)
	- direct fluorescent antibody testing for T pallidum: nr (%)	11 (52%)
	- dark-field microscopy: nr (%)	7 (33%)
	- Warthin-Starry staining: nr (%)	3 (14%)
	- Dieterle staining: nr (%)	1 (5%)
	FNAB of lymph-node: nr (%)	12 (22%)
	Lymph-node biopsy: nr (%)	2 (4%)
	Treponemal test: nr (%)	25 (45%)
	- TPHA: nr (%)	19 (76%)
	- FTA-ABS: nr (%)	11 (44%)
	Non-treponemal test: nr (%)	32 (58%)
	- RPR: nr (%)	23 (72%)
	- VDRL: nr (%)	8 (25%)
	- TRUST: nr (%)	1 (3%)
Radiological examinations	CT: nr (%)	12 (22%)
	PET: nr (%)	2 (4%)
	US: nr (%)	1 (2%)
Radiological signs	High enhancement	12 (86%)
	Cystic and/or necrotic areas	6 (43%)
	Hypertrophic tissue	3 (21%)
	Ulcerous lesions	3 (21%)

* = high-risk sexual practices, multiple sex partners and the absence of condoms during sexual contacts

**Table 4:** Comparison between clinical manifestation and signs of primary, secondary and third syphilis

Variables	Patients	Primary syphilis	Secondary syphilis	Third syphilis
Ulcerous lesion	27 (45%)	14 (56%)	10 (36%)	3 (43%)
Tissue hypertrophy	13 (22%)	7 (28%)	6 (21%)	0 (0%)
Mucosal patch	8 (13%)	2 (8%)	6 (21%)	0 (0%)
Hyperaemia	5 (8%)	2 (8%)	3 (11%)	0 (0%)
Gumma	4 (7%)	0 (0%)	0 (0%)	4 (57%)
Leukoplakia	3 (5%)	0 (0%)	3 (11%)	0 (0%)
Total	60	25	28	7

### Laboratory test, histological features and radiological images of oropharyngeal syphilis

Nowadays, multiple methods are available for the laboratory detection and histological diagnosis of syphilis. However, the laboratory diagnosis of syphilis remains predominantly serological. There are two different types of serologic tests based on the type of antigen the antibodies are directed against: treponemal and non-treponemal tests. The current cohort of patients underwent treponemal tests in 25 (45%) cases, such as Treponema Pallidum Hemagglutination (TPHA) test (19 cases) and Fluorescent Treponemal Antibody Absorbed (FTA-ABS) test (11 cases). On the other hand, non-treponemal tests were used in 31 (55%) cases, such as Rapid Plasma Reagin (RPR) test (23 cases), Venereal Disease Research Laboratory (VDRL) test (8 cases), and the Toluidine Red Unheated Serum Test (TRUST) (1 case). A combination of treponemal methods and non-treponemal assays to determine disease activity was used in 80% patients. However, another feasible way to diagnose Treponema pallidum in oropharynx was direct bacterial detection through a surgical biopsy of pharyngeal tissue. Such a procedure was used in 21 patients (38%) and spirochetes were detected through direct fluorescent antibody testing for T pallidum (11 cases), dark-field microscopy (7 cases), Warthin-Starry staining (3 cases) and Dieterle staining (1 case). An enlargement of a singular or multiple lymph node(s) was reported in 14 cases. Twelve patients underwent a fine needle biopsy (FNAB) and 2 patients’ surgical biopsies of lymphadenopathies. In conclusion, a total amount of 46 (83%) patients was undergone to serological tests and 30 (54%) patients were undergone to tissue examination. Moreover, 19 (34%) cases were diagnosed using both serological and tissue methods.

Radiological tools employed to examine lesions and to stage patients were computed tomography (CT), positron emission tomography (PET) and ultrasound (US) examination, which were used in 12, 2 and 1 patient, respectively. The most frequent radiological sign was contrast enhancement of the lesion (82%), followed by cystic or necrotic areas (43%), hypertrophic tissue (21%) and ulcerous lesions (21%).

## Discussion

The main risk factors related to syphilitic infection were MSM (49% patients of the current cohort), smoke, drug misuse and HIV [8, 11]. Syphilis can disproportionately affect MSM due to high-risk sexual behavior, including high-risk sexual practices, multiple sex partners and the absence of condoms during sexual contacts, and concurrent use of abuse substances [12]. Syphilis was also associated with the frequency, duration and status of tobacco use and with HIV co-infection [13, 14]. Serological tests are considered the gold standard for screening, diagnosing and monitoring disease activity, consisting of treponemal and non-treponemal tests [9]. Treponemal tests are qualitative assays performed on serum, to detect antibodies against a variety of luetic antigens [15]. Conversely, non-treponemal tests are quantitative methods, performed on serially diluted serum to detect total antibodies directed against lipoidal antigens, such as cardiolipin and lecithin, which are released from damaged host cells and the bacteria [16]. Satyaputra et al. [9] described two different syphilis tests algorithms for syphilis diagnosis. The first one is called “traditional algorithm”, using non-treponemal assay for primary evaluation, followed by a confirmatory treponemal assay for reactive samples only. As non-treponemal assays are less sensitive in early and latent disease, there is a potential risk of under diagnosis [17]. Therefore, a treponemal test should be performed when suspecting these stages of infection despite a non-reactive non-treponemal test. Conversely, the “reverse algorithm” firstly uses a treponemal assay followed by a non-treponemal assay for reactive samples [9]. When a new diagnosis of syphilis is made or if a discordant result is obtained, a second treponemal assay, using a different platform is performed for confirmation [18]. Diagnosis or exclusion of syphilis, however, requires collective results from both treponemal and non-treponemal tests in most suspected cases with a high clinical probability [19]. Our systematic revision evidenced that 80% of patients underwent both treponemal and non-treponemal laboratory testing. As a consequence, there was a high rate of oropharyngeal biopsies or lymph node FNAB in patients without previous treponemal and/or non-treponemal test (50% versus 27%), likely caused by difficult-to-recognize oropharyngeal syphilitic lesions, and low awareness of attending physicians, that may well have a low suspicion rate of head and neck syphilis. The detection of Treponema pallidum in oropharyngeal tissue was detected direct fluorescent antibody testing for T pallidum, dark-field microscopy, Warthin-Starry staining and Dieterle staining. However, dark-field microscopy is not recommendable for diagnosing syphilis in oropharyngeal cases due to the high-risk presence of saprophytic treponemas can be present. A recent study reported report results of PCR testing for *Treponema pallidum* DNA from oropharyngeal swabs, blood and oropharyngeal lesion exudate swabs from individuals with early syphilis [20]. *Treponema pallidum* DNA was amplified from 26,8% and 26,7% of oropharyngeal swabs and blood test, respectively, from individuals with all syphilis stages, including two individuals with nonreactive serum test [20]. *Treponema pallidum* DNA was identified in 41% of individuals with syphilis in whom both an oropharyngeal swab and blood were tested [20]. Therefore, amplification tests can identify recent *Treponema pallidum* infection and may be particularly useful for diagnosis of very early or asymptomatic syphilis. Moreover, a comprehensive guidance on clinical recognition of syphilis, diagnostic-test algorithms and recommended treatment regimens, recently proposed by Ghanem et al. [21], reported that PCR testing is worldwide recognized as one of the most sensitive and specific direct testing options in syphilitic lesions.

The most common locations for the chancre of primary syphilis are genitals, followed by oral cavity [22–25]. Kahn et al. [8] reviewed clinical manifestations of head and neck syphilis, showing how oral cavity was the most common involved site, in particular labial commissure (32%) and tongue (24%). Conversely, the literature reported how oropharyngeal is considered an uncommon site of infection. Therefore, tonsil is the most frequent site of oropharyngeal syphilis [25]. To support this evidence, our analysis reported how tonsil was the most implicated site of oropharynx (71%). In line with our findings, Hamlyn et al. [26] underlined how patients affected by oropharyngeal syphilis revealed multiple lesions, in the form of mucous patches and painful, serpiginous ulcerous lesions within the oral cavity and pharynx, which may extend onto the surface of the tonsil. According with the stage of syphilis, different types of lesions have been described in the head and neck area (Table 4). Our analysis showed that patients affected by primary oropharyngeal syphilis presented with ulcerous lesions (45%), tissue hypertrophy (22%) and mucosal patches (13%). Mucosal infection of head and neck is indeed diagnosed mainly during the second phase of disease [15]. Oral cavity secondary syphilis was reported as aphthous, pseudomembranous lesions or leukoplakia [17, 19]. Accordingly, our systematic revision confirmed that nearly half (48%) of patients affected by oropharyngeal syphilis were diagnosed in their secondary syphilis, manifesting with ulcerous lesions (56%) and tissue hypertrophy (28%). Indeed, these patients usually also exhibited a generalized rash, helping physicians to the right diagnosis [27]. Tertiary syphilis typically presents with nodular or gumma’s lesions, often involving also hard and soft palate [8, 28, 29]. These lesions are easily mistaken with a variety of other diseases, including discoid lupus erythematous, atypical mycobacterial infections and sarcoidosis [30]. Our systematic analysis showed that syphilis gumma’s lesions and ulcerous lesions occurred in 57% and 43% of cases, respectively, in patients affected by tertiary syphilis. Cervical lymphadenopathies were frequently associated with oropharyngeal syphilis, calling for differential diagnosis with head and neck cancer as OPSCCs, lymphomas and other neoplastic conditions sharing similar clinical and radiological features. Luetic lymphadenitis was characterized by painless and firm enlarged lymph nodes, more frequently found as a single node in patients in their primary syphilis, or multiple and generalized lymphadenopathies, which characterized patients in their secondary syphilis [31, 32]. Indeed, lymph nodes were usually characterized by cystic signs on US, necrotic or cystic-appearing on CT and MRI and nodal increased contrast enhancement in CT, MRI and PET-FDG [33–35]. Such aspects often raise concerns for malignancy, as regional metastases of OPSCC can also exhibit a cystic and necrotic core of the lymph nodes [35]. As a consequence, suspect lymph nodes warrant further workup with FNAB, which has an excellent sensitivity and specificity for detecting oropharyngeal or laryngeal malignancy [36–38]. In the present series, 14 cases of cervical neck lymph nodes were described 12 of them underwent FNAC and 2 surgical biopsies. In particular, 60% of patients with suspicious lymphadenopathies underwent an invasive procedure without previous laboratory tests. Moreover, few cases of oropharyngeal syphilis were detected through both invasive procedure and laboratory tests, to remark the difficulty to make an a-priori prediction of oropharyngeal syphilitic lesions. Histopathological features included marked follicular hyperplasia, occasionally mimicking follicular lymphoma, extensive plasma cell proliferation, non-caseating epithelioid granulomas, multinucleated giant cells, fibrosis and inflammation of the capsule, as well as arteritis and phlebitis of newly formed vessels with hyperplasia of endothelial cells [39–41]. The same histological findings were observed during examination of samples of oropharyngeal tissue (38% of patients).

Direct detection methods, such as molecular or specialized microscopy techniques, were used to search for treponemas, but use of such assays is frequently limited by their poor performance, restricted availability and cost [9]. Direct fluorescent antibody testing for Treponema pallidum is the most used method (52%). Silver and histological stains, as the Steiner or Warthin-Starry stain, can be utilized to highlight spirochetes in formalin-fixed, paraffin-embedded tissue from lesions of primary and secondary syphilis [41]. Compared to the silver stain, immunohistochemistry offers better sensitivity and specificity [42].

Effective and early clinical recognition of oropharyngeal syphilitic lesions and prompt execution of both treponemal and non treponemal laboratory tests can help avoiding histological examination of surgical biopsies through open, microscopic or robotic methods [43, 44]. At the same time, pathologists have to investigate about the detection of syphilitic microorganism in oropharyngeal tissue sampled for a suspicious for OPSCC [9].

## Limitations and future perspectives

**Fig. 1:**
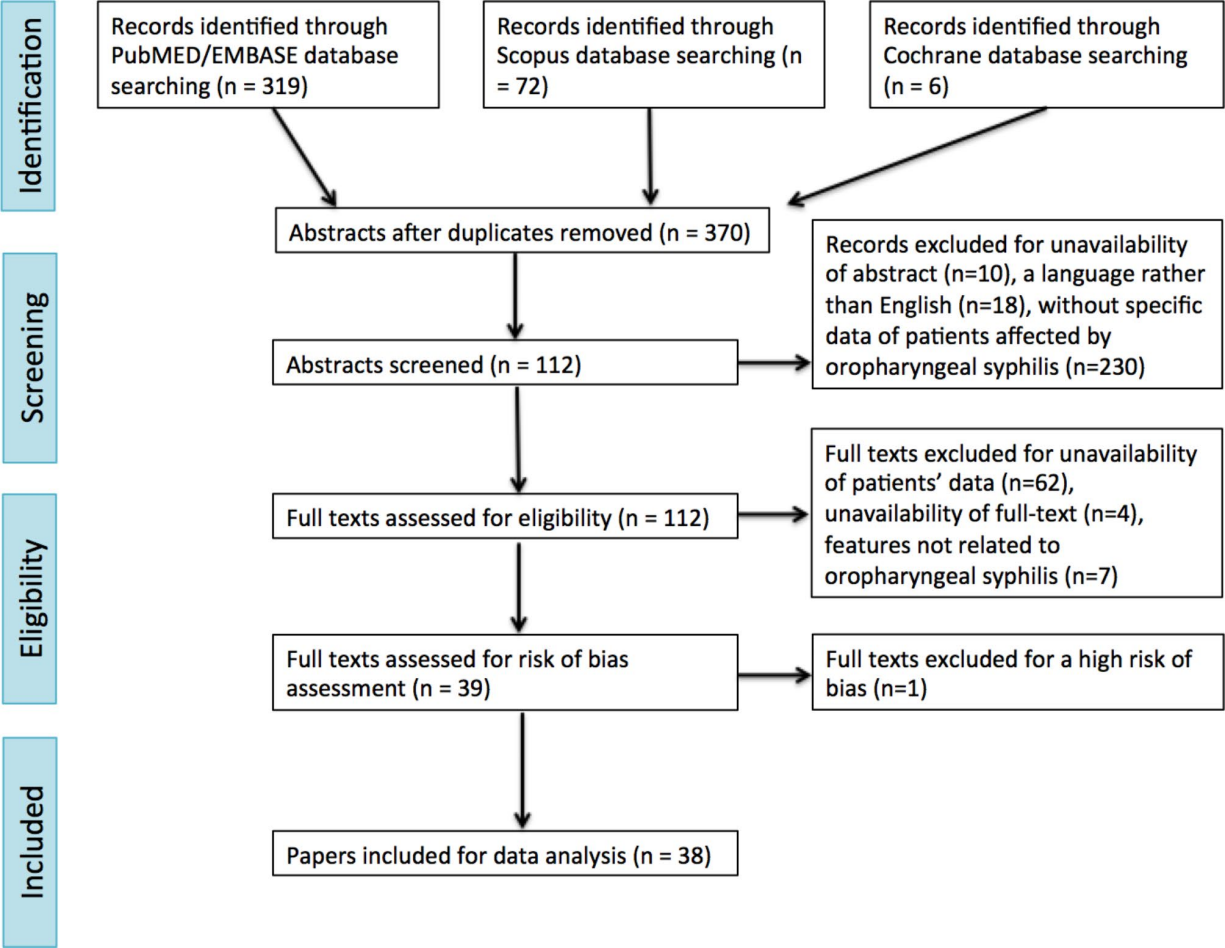
Flow chart of the manuscript

The systematic review reported in this paper is the first one, to our best knowledge, focusing exclusively on oropharyngeal syphilis and related head and neck adenopaties. The literature investigated includes many case reports and a few case series of patients. Many studies do not show exhaustive information about clinical presentation, symptoms and signs, histological features, laboratory tests and radiological findings of affected patients. For this reason, the Authors were led to exclude such papers for the analysis. However, it is important to acknowledge that the included articles cover a period of 50 years, during which the diagnostic criteria for syphilis underwent constant changes and varied across different regions. Consequently, the homogeneity of the included studies was limited. Nevertheless, our results can be deemed interesting to enable an exhaustive overview of clinical and histological features of oropharyngeal syphilis (Fig. 1).

## Conclusions

Syphilis infection rates are on the rise, with an increasing number of patients often manifesting head and neck luetic signs. Syphilis has been historically referred to as the “great imitator”, as its manifestations may be similar to those of malignances, such as OPSCC or lymphomas. Consequently, in patients exhibiting non-specific oropharyngeal lesions suggesting malignancy, a screening for syphilis should also be performed. An overview of clinical manifestations of oropharyngeal luetic infections, that is how and where syphilis may affect oropharynx, may help physicians in early suspicion as well as in early diagnosis.

## Data Availability

All data generated or analyzed during this study are included in the published article.
